# Olefin Recovery by *BEA‐Type Zeolite Membrane: Affinity‐Based Separation with Olefin−Ag^+^ Interaction

**DOI:** 10.1002/asia.202100096

**Published:** 2021-03-18

**Authors:** Motomu Sakai, Yuto Tsuzuki, Naoyuki Fujimaki, Masahiko Matsukata

**Affiliations:** ^1^ Research Organization for Nano & Life Innovation Waseda University 513 Waseda-Tsurumaki-cho, Shinjuku-ku Tokyo 162-0041 Japan; ^2^ Department of Applied Chemistry Waseda University. 513 Waseda-Tsurumaki-cho, Shinjuku-ku Tokyo 162-0041 Japan; ^3^ Advanced Research Institute for Science and Engineering Waseda University 513 Waseda-Tsurumaki-cho, Shinjuku-ku Tokyo 162-0041 Japan

**Keywords:** zeolite, membrane separation, *BEA, ethylene, propylene

## Abstract

Ag^+^ was introduced into *BEA‐type zeolite membrane by an ion‐exchange method to enhance olefin selectivity. Ag−*BEA membrane exhibited superior olefin separation performance for both ethylene/ethane and propylene/propane mixtures. Particularly, the separation factor for ethylene at 373 K reached 57 with the ethylene permeance of 1.6×10^−7^ mol m^−2^ s^−1^ Pa^−1^. Adsorption properties of olefin and paraffin were evaluated to discuss contribution of Ag^+^ to separation performance enhancement. A strong interaction between olefin and Ag^+^ in the membrane caused preferential adsorption of olefin against paraffin, leading to selective permeation of olefin. Ag−*BEA membrane also exhibited high olefin selectivities from olefin/N_2_ mixtures. The affinity‐based separation through Ag−*BEA membrane showed a high potential for olefin recovery and purification from various gas mixtures.

## Graphical abstract

Ag−*BEA membrane for olefin recovery: Ag^+^ was introduced into *BEA‐type zeolite membrane. We found that such a membrane exhibited superior ethylene and propylene selectivities. Ag−*BEA membrane had a high potential for olefin recovery from various gas mixtures.

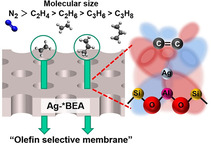

Membrane separation is drawn attention as a novel energy‐saving processes for a wide variety of chemical separation such as propylene and ethylene purification. Purifications of propylene and ethylene consumes as much as 0.3% of global energy use.^[1]^ Sholl and Lively pointed out that membrane separation and membrane‐distillation hybrid process should be introduced to alkene purification from alkene/alkane mixture to reduce the energy consumption.^[1]^ In addition, membrane‐distillation hybrid processes has potential for 10–50% of operating cost reduction compared with conventional cryogenic distillation.[^2^, ^3^, ^4^] For these environmental and economical advantages, various types of olefin selective membranes have been investigated for the last decades.

Propylene/propane or ethylene/ethane separation based on molecular sieving effect has previously been attempted by using porous inorganic membranes such as carbon molecular sieve (CMS),[^5^, ^6^, ^7^, ^8^] silica,^[9]^ and metal organic framework (MOF).[^10^, ^11^, ^12^] Inorganic membranes are expected to have high chemical resistance and mechanical strength, and thus have become recognized as candidates for olefin separation that was generally operated at elevated pressure. In these inorganic membranes, ethylene (or propylene) preferentially penetrates through membranes because its molecular size is slightly smaller than that of ethane (or propane).

Ethylene and propylene selective Ag^+^‐containing organic polymer membranes have been also reported. Ag^+^‐containing polymeric membranes showed the superior separation performance by strong interaction between Ag^+^ and olefins.[^13^, ^14^, ^15^] Ag^+^ in facilitated transport membranes plays as carrier for the transport of olefins. Although possessing great advantage in separation performance, these Ag^+^‐containing polymeric membranes have difficulty in stability owing to their weak chemical and mechanical strength. In addition, liquid phase‐facilitated transport membrane exhibited high olefin selectivity only in the presence of water and readily lose its selectivity by the leakage of carrier.

Since zeolite can occlude cation in its micropore as exchanged ion, we have studied Ag^+^‐exchanged zeolite as membrane material. Both characters of high stability in inorganic membrane and high selectivity in Ag containing polymeric membrane are expected for Ag^+^‐exchanged zeolite membrane. Previously we reported Ag−X membrane having a good propylene/propane separation performance, separation factor of 55.4 for propylene/propane mixture at 353 K.^[16]^ In Ag−X membrane, propylene adsorbed predominantly by strong interaction with Ag^+^ and blocked propane permeation, resulting in that propylene preferentially penetrated through Ag−X membrane. Propane permeance through Ag−X membrane in the binary system was two order of magnitudes smaller than that in the unary system owing to the blocking by propylene. In Ag−X membrane, the adsorption selectivity for propylene mainly dominated the permeation selectivity.^[17]^

*BEA‐type zeolite which is a kind of large pore zeolites similar to X‐type zeolite and is often used as catalyst. We recently developed a defect‐less *BEA membrane by a unique method without using organic structure‐directing agent (OSDA).^[18]^ The *BEA membrane synthesized under OSDA‐free conditions had a specific feature, large ion‐exchange capacity. We expect that such *BEA membrane having large ion‐exchange capacity is suitable membrane material for olefin separation. In this study, we prepared Ag−*BEA membrane and investigated the olefin separation and permeation performance.

Figures 1(a) and (b) shows the photos of Na−*BEA and Ag−*BEA membrane prepared, respectively. The Ag−*BEA membrane was prepared by ion‐exchange with AgNO_3_ aqueous solution for Na−*BEA. By ion‐exchange, the color of membrane surface changed from white to light gray. A thin and compact layer of Ag−*BEA crystals synthesized on the outer surface of support are observed in typical FE‐SEM images, as shown in Figures 1(c) and (d).

**Figure 1:**
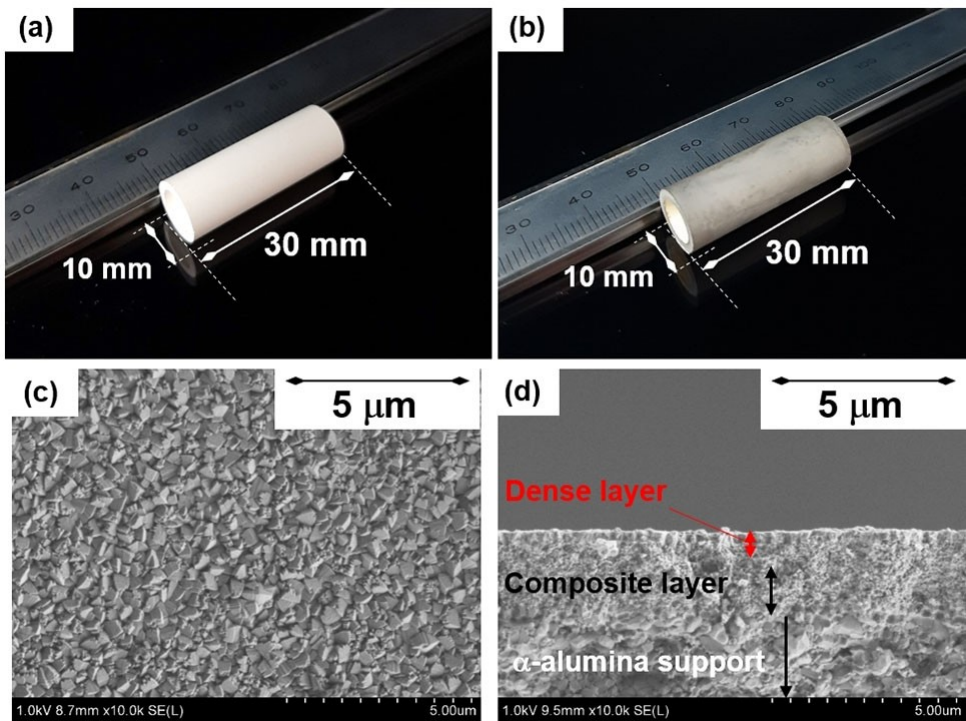
Photos of (a) Na‐, (b) Ag−*BEA membrane synthesized on α‐alumina support. Typical FE‐SEM images of (c) surface and (d) cross‐section of Ag−*BEA membrane.

We investigated the effect of Ag^+^ introduction on the permeation and separation properties. Figure 2 shows the results of separation tests for an equimolar ethylene/ethane mixture through (a) Na‐ and (b) Ag−*BEA membranes. The ethane permeance markedly decreased to less than a tenth by changing cation from Na^+^ to Ag^+^, resulting in that the separation factor of ethylene/ethane drastically increased from around 2 to above 60 by the ion‐exchange. For example, the separation factor for ethylene through Ag−*BEA membrane at 333 K was 77.1 with its permeance of 1.04×10^−7^ mol m^−2^ s^−1^ Pa^−1^. The ethylene permeances through both membranes increased with increasing temperature, possibly because the diffusivities of ethylene in these membranes improved at elevated temperature.

**Figure 2:**
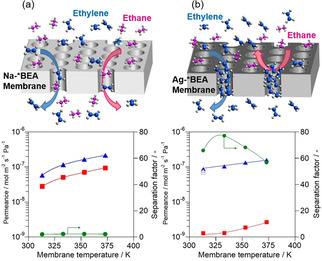
Results of separation tests for ethylene/ethane equimolar mixture through (a) Na‐, (b) Ag−*BEA membrane. ▵, ethylene; □, ethane; ○, separation factor. Closed symbol, binary system; open symbol, unary system.

Figure 2 compares the permeances in the unary and binary systems. The permeances of ethylene and ethane in the unary systems at 313 K through Ag−*BEA membrane were plotted as open symbols in Figure 2 (b): The ethane permeance in the unary system was 10 times larger than that in the binary system. This phenomenon is similar to that observed with Ag−X membrane as described above.^[17]^ We consider that the remarkable reduction of ethane permeance in the binary system was caused by the filling of zeolite micropore with ethylene, as schematically drawn in Figure 2 (b).

It is noted that the separation ability of Ag−*BEA for the ethylene/ethane mixture was much superior to that of Ag−X membrane which we previously reported, 15.9 at 303 K.^[16]^ A relatively smaller pore size of *BEA‐type zeolite, 0.66 nm, may contribute to blocking of ethane permeation by ethylene compared with that of X‐type zeolite, 0.74 nm.

Figure 3 shows the separation properties of (a) Na‐, (b) Ag−*BEA membranes for an equimolar propylene/propane mixture. The permeation behaviors of propylene and propane were almost the same as those observed in the ethylene/ethane separation. The propylene permeance through Ag−*BEA membrane was several times smaller than that of ethylene: The separation factor at 373 K was 82.9 with the propylene permeance of 3.57×10^−8^ mol m^−2^ s^−1^ Pa^−1^. Ethylene would have a larger diffusivity in micropore because of its small size compared with propylene, resulting in its larger permeance.

**Figure 3:**
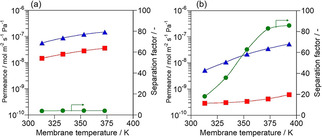
Results of separation tests for propylene/propane equimolar mixture through (a) Na‐, (b) Ag−*BEA membrane. ▵, propylene; □, propane; ○, separation factor.

We herein studied the adsorption properties of ethylene, ethane, propylene, and propane on Ag−*BEA membrane, as shown in Figure 4. Each isotherm was evaluated at 313 K in the unary systems. To obtain precise isotherms, a sample holder and measurement equipment that we specially designed enabled us to insert the whole membrane without destruction with a minimized dead‐volume, leak, and accurate control of temperature.^[17]^ It is noteworthy that the adsorbed amounts of ethylene and propylene on Ag−*BEA membrane markedly increased at very low pressures at around 10^−3^ kPa.

**Figure 4:**
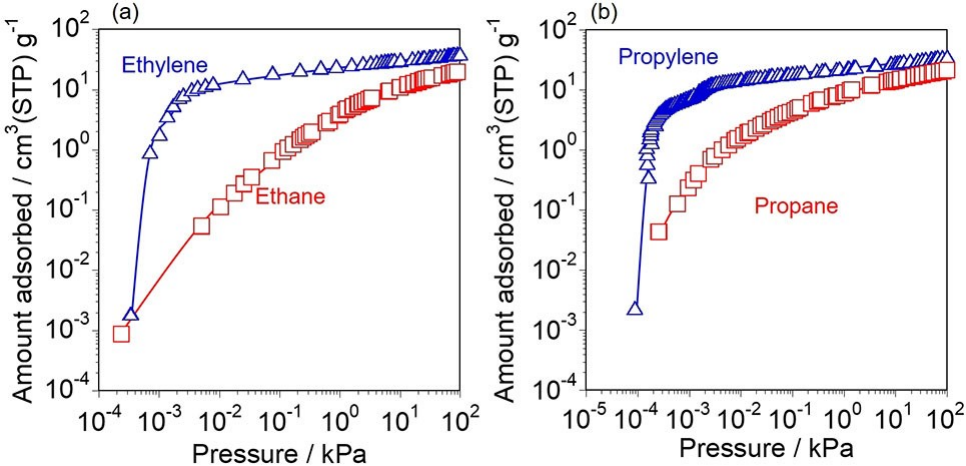
Adsorption isotherms on Ag−*BEA membrane at 313 K. (a) ▵, ethylene and □, ethane. (b) ▵, propylene and □, propane.

The adsorption equilibrium constants, *K* (Pa^−1^) were calculated from the isotherms according to the Langmuir's equation as follows.

(1) $$P\;\cdot \;V{}^{-1}=P\;\cdot \;Vs{}^{-1}+K{}^{-1}\;\cdot \;Vs{}^{-1}$$

where *P* is the pressure (Pa), *V* is the amount adsorbed (cm^3^(STP) g^−1^), and *V_S_* is the saturated adsorption amount (cm^3^(STP) g^−1^). Therefore, the values of *V_S_* and *K* are calculated from the slope and the intercept of *P*⋅*V* ^−1^ vs. *P* plot. As a result, *K_propylene_* (1.78) and *K_ethylene_* (0.845) were much greater than *K_propane_* (0.475) and *K_ethane_* (0.188).

We considered that Ag−*BEA membrane showed the high olefin selectivity based on such differences of affinity with Ag^+^ between olefin and paraffin. As in the case of Ag−X membrane, adsorption of olefins on Ag−*BEA membrane play an important role for expression of olefin selectivity.

As shown in Figures 2 and 3, Ag−*BEA membrane exhibited superior separation performance for both ethylene/ethane and propylene/propane mixtures. Although many studies about molecular sieving membranes for the propylene/propane or ethylene/ethane separation have been reported as described above, these molecular sieving membranes have shown a high selectivity for either ethylene or propylene. Suitable pore sizes by molecular sieving are different for ethylene/ethane and propylene/propane separation. In other words, individual membranes have to be used for each separation system. For example, it was reported that a separation factor of ZIF‐8 membrane for propylene/propane exceeded 100,^[10]^ whereas that for ethylene/ethane was only 2.0,^[11]^ suggesting that ZIF‐8 is a promising membrane material for propylene/propane separation by size. Its pore size is, however, too large to separate ethylene/ethane.

We compared the performance of Ag−*BEA membrane to those of other (a) propylene[^9^, ^10^, ^19^, ^20^, ^21^, ^22^, ^23^, ^24^, ^25^, ^26^] and (b) ethylene[^5^, ^6^, ^7^, ^8^, ^11^, ^27^, ^28^, ^29^] selective inorganic membranes previously reported, as shown in Figure 5. These Robeson plots clearly show that Ag−*BEA membrane is promising owing to the larger permeance and superior selectivity to olefin for the separation of olefin/paraffin mixture. In particular, the separation and permeation properties of Ag−*BEA membrane for ethylene/ethane overwhelms those of other membranes. The ethylene permeance was four orders of magnitude larger than that those through membranes showing similar separation factors like CMS; The separation factor was about 30‐times greater than those through membranes showing the subequal permeances like ZIF‐8.

**Figure 5:**
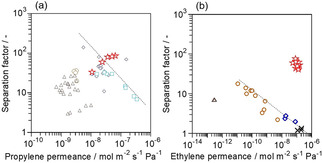
Robeson plots of (a) propylene/propane and (b) ethylene/ethane separations. ○, CMS; ◊, ZIF‐8; □, silica; ▵, mixed matrix; ×, alumina; ☆, Ag−*BEA (this study).

We would like to propose a possible new purification process for olefin production by using olefin‐selective Ag−*BEA membrane. Currently, ethylene and propylene were purified from a mixture of C_1_−C_4_ hydrocarbons by a series of distillations^[30]^ (shown in Figure S1(a) in supporting information). In this process, methane was removed by the first distillation tower, and then ethylene and ethane were separated from the remained C_2_−C_4_ mixture. C_4_ hydrocarbons were, then, separated from C_3_−C_4_ mixture. The obtained mixture of ethylene/ethane and propylene/propane were fed to distillation towers for ethylene and propylene purifications, respectively. These distillation towers for ethylene and propylene purification consume most of the energy of the whole purification system mainly because of a small deference of boiling points at low temperature and a high reflux ratio (Δb.p. of ethylene and ethane, 15 K; Δb.p. of propylene and propane, 5.6 K).

Taking the advantage of affinity‐based olefin separation, we would like to propose a novel olefin purification process to reduce the energy cost and number of distillation tower (shown in Figure S1(b) in supporting information). After removing methane in the first distillation tower, the mixture of C_2_−C_4_ is fed to a membrane unit for separating olefins and paraffins. In this system, a mixture of ethylene, propylene, and butenes would be recovered from the permeate side and that of ethane, propane, and butanes remained in the retentate side, respectively. Each mixture of C_2_−C_4_ which have large differences of boiling points (Δb.p. of ethylene and propylene, 56 K; Δb.p. of propylene and 1‐butene, 41 K) can be separated easily by a distillation with low energy consumption.

Figure 6 shows the separation performance of Ag−*BEA membrane for an equimolar mixture of ethylene/ethane/propylene/propane. As expected, both propylene and ethylene selectively permeated from the ternary mixture through Ag−*BEA membrane.

**Figure 6:**
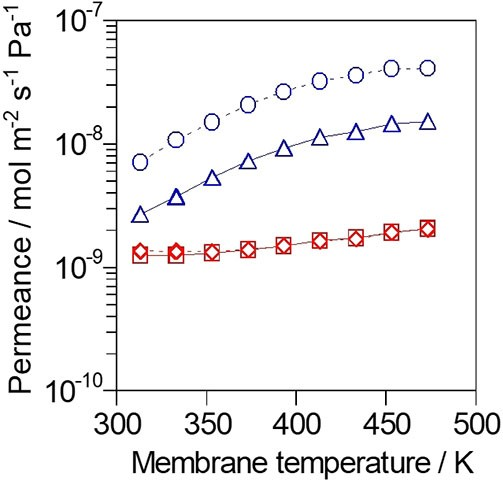
Results of separation tests for ethylene/ethane/propylene/propane equimolar mixture through Ag−*BEA membrane. ▵, ethylene; □, ethane; ○, propylene; ◊, propane.

Affinity‐based membrane separation found in this study would have a pottential to innovate the purification processes in olefin production process such as naphsa cracking and fluid catalytic cracking. The separation property shown in Figure 6 suggeted that olefin purity in the permeate reached above 93% from 50% in the feed.

In addition, we found that Ag−*BEA membrane exhibited high olefin selectivities in olefin/N_2_ separation tests (olefin/N_2_=15/85 kPa), as shown in Figure 7. Whereas N_2_ is smaller than both ethylene and propylene, Ag−*BEA membrane showed excellent ethylene and propylene selectivities of 106 at 333 K and 181 at 393 K, respectively. In polymerization plant, unreacted monomers such as ethylene and propylene are removed from polymers by purge gas, nitrogen. The vent gas contains around 10–20 vol % of monomer in nitrogen. The value of unrecovered monomers reached a million dollar annually at a typical polymerization plant.^[31]^ Although some recovery process using rubbery polymeric membrane have been proposed, separation performances of silicone rubbery membranes (C_2_^=^/N_2_=6.3, C_3_^=^/N_2_=16.2) were not sufficient.^[32]^ In our elementary calculation, about seven‐tenths of propylene purged could be recovered with 95% of purity by using Ag−*BEA membrane with separation factor of 180 (shown in Figures S2 and S3). Olefin recoveries from purge gas in olefin polymerization processes would also be the targets to apply Ag−*BEA membrane to.

**Figure 7:**
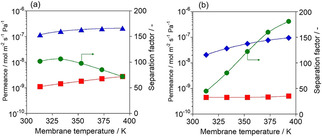
Results of separation tests for olefin/N_2_ mixture through Ag−*BEA membrane. (a) C_2_^=^/N_2_. (b) C_3_^=^/N_2_. ▵, olefins; □, N_2_; ○,separation factor.

As described above, the principle of affinity‐based separation can apply to wide separation targets. Ag−*BEA membrane is a prospective material for olefin recovery from various gas mixture irrespective of each molecular size.

## Experimental Section

OSDA‐free *BEA membrane, containing Na^+^ as counter cation, was prepared by a seed assisted method. The synthesis procedure was described elsewhere.^[18]^ OSDA‐free Na−*BEA membrane formed on the outer surface of support. A porous tubular α‐alumina (o.d.=10 mm, i.d.=7 mm, length=30 mm, average pore size=150 nm) was used as a support.

Ag−*BEA membrane for propylene/propane and ethylene/ethane separation was prepared by the ion‐exchange of OSDA‐free Na−*BEA membrane with 10 mM of silver nitrate aqueous solution. The membrane was immersed in AgNO_3_ aqueous solution for 1 h at 353 K while stirring. Then, *BEA membrane was washed with distilled water and dried at 343 K overnight prior to use.

Permeation and separation properties for olefin/paraffin mixtures were evaluated, as follows. A mixture of ethylene/ethane, propylene/propane, propylene/N_2_, ethylene/N_2_, or ethylene/ethane/propylene/propane was fed to the outer surface of tubular support. The permeate side, the inside of tubular support, was swept with argon and both of feed and permeate sides were kept at atmospheric pressure.

In the permeation and separation measurements, the permeation flow rate was determined by a gas chromatography equipped with a flame ionization detector (GC‐FID, GC‐8A, Shimadzu) by using internal standard gas, methane. Permeation flux, *J*, was calculated as follows.

(2) $$J{}_{X}\;\left(\mathrm{mol}\;m{}^{-2}\;s{}^{-1}\right)=u{}_{X}\;A{}^{-1}$$

*u_X_* is the flow rate (mol s^−1^) of component *X* and *A* is the membrane area (m^2^). And then, permeance, *Π*, and separation factor *α*_*X/Y*_ were determined using the following equations (3) and (4);3, 4

(3) $$\Pi {}_{X}\;\left(\mathrm{mol}\;m{}^{-2}\;s{}^{-1}\;\mathrm{Pa}{}^{-1}\right)=J\;\Delta p{}_{X}{}^{-1}$$

(4) $$\alpha {}_{X/Y}\;\left(-\right)=Y{}_{A}\;Y{}_{B}{}^{-1}\;X{}_{A}{}^{-1}\;X{}_{B}$$

where *X*_A_ and *X*_B_ are molar fractions of components A and B in the feed. *Y*_A_ and *Y*_B_ are molar fractions of components A and B in permeate, respectively.

## Conflict of interest

The authors declare no conflict of interest.

## Supporting information

As a service to our authors and readers, this journal provides supporting information supplied by the authors. Such materials are peer reviewed and may be re‐organized for online delivery, but are not copy‐edited or typeset. Technical support issues arising from supporting information (other than missing files) should be addressed to the authors.

Supplementary
