# Supplementary material for: Olefin Recovery by *BEA‐Type Zeolite Membrane: Affinity‐Based Separation with Olefin−Ag+ Interaction
Source: Chem Asian J. 2021 Mar 18;16(9):1101–5. doi: 10.1002/asia.202100096 (PMC8251837; doi:10.1002/asia.202100096)
Supplement: Supplementary file 1 — Supplementary [file ASIA-16-1101-s001.pdf]

CHEMISTRY
AN ASIAN JOURNAL

Supporting Information

Olefin Recovery by *BEA-Type Zeolite Membrane: Affinity-Based Separation with Olefin–Ag^+ Interaction

Motomu Sakai,* Yuto Tsuzuki, Naoyuki Fujimaki, and Masahiko Matsukata© 2021 The Authors. Chemistry - An Asian Journal published by Wiley-VCH GmbH. This is an open access article under the terms of the Creative Commons Attribution License, which permits use, distribution and reproduction in any medium, provided the original work is properly cited.

Experimental Procedures

Membrane Preparation
OSDA-free *BEA membrane, containing Na^+ as counter cation, was prepared following our previous research.[1]
A support was immersed into seed slurry to load seed crystal for 1 min then dried at 343 K for 2 h. The seeded support was calcined at 803 K for 6 h to remove OSDA in micropore of seed crystals.
According to a previously reported procedure,[2] the synthesis gel having a composition of $100SiO_2$:$1Al_2O_3$:$30Na_2O$:$2000H_2O$ was prepared by mixing of colloidal silica, sodium hydroxide, sodium aluminate and distilled water. Colloidal silica was poured in a drop-wise manner to a mixture of the other chemicals. Aging was taken place for the resulting mixture at 333 K for 4 h prior to crystallization.
Following calcination, the seeded support was settled into a PTFE-lined autoclave with the synthesis gel (40 g) and crystallized at 393 K for 7 days under static condition. After crystallization, obtained membrane was washed with boiling water and dried overnight.
Ag-*BEA membrane was prepared by an ion-exchange method. Na-*BEA membrane synthesized was immersed into silver nitrate aqueous solution and kept while stirring for 1 h at 353 K. The ion-exchanged *BEA membrane was washed with distilled water and dried at 343 K overnight prior to use.

Adsorption measurement
Adsorption measurement of olefin and paraffin on Na- and Ag-*BEA membranes were carried out a non-destructive adsorption method. The measurement was taken place by using a BELSORP-max (MicrotracBEL Corp.) instrument with a special sample holder developed in-house. The homemade sample holder gives us a permission to insert the whole of membrane without destruction. The membrane sample was outgassed at 373 K for 36 h under vacuum prior to the adsorption tests. Adsorption measurements were performed at 313 K.

Chemicals and Materials
A porous tubular α-alumina (o.d. = 10 mm, i.d. = 7 mm, length = 30 mm, average pore size = 150 nm, Noritake Co. Ltd.) was used as the support.
Colloidal silica (ST-S, Nissan Chemical).
Sodium hydroxide (> 97 %, Kanto Chemical).
Sodium aluminate (Na_2O, 31.0-35.0%; Al_2O_3, 34.0-39.0%, Kanto Chemical).
Silver Nitrate (99.8%, Wako Pure Chemical, Ltd.)

Results and Discussion

Olefin purification system from C_2-C_4 mixture
Fig. S1 shows (a) conventional process and (b) novel process including olefin concentration membrane for olefin purification from C_2-C_4 hydrocarbon mixture.

Calculation for $C_3^=$/N_2 separation with Ag-*BEA membrane
Fig. S2 shows a model of calculation for $C_3^=$/N_2 mixture separation using Ag-*BEA membrane. Membrane unit was divided as series of micro cell, as shown in Fig. S2. At first, feed stream ($C_3^=$/N_2 = 15/85 mol%) was inlet to the first cell. Permeation flow rates of $C_3^=$ and N_2 in the first cell were calculated according to equation (1). And then, retentate flow rates of $C_3^=$ and N_2 in the first cell were calculated according to equation (2). The retentate flow rate became the feed flow rate of the second cell.

P_X (mol s^{-1}) = Π_X x A x ΔC_X (1)

F_X (mol s^{-1}) = $P_X + R_X$ (2)

Here, F_X, P_X, R_X, Π_X are feed flow rate (mol s^{-1}), permeate flow rate (mol s^{-1}), retentate flow rate (mol s^{-1}), and permeance of component X (mol m^{-2} s^{-1} Pa^{-1}), respectively. A is membrane area (m^2). ΔC_X is a partial pressure difference of component X between permeate and retentate side (Pa).

Feed and membrane conditions used in this calculation are fixed as follows.
Feed flow rate; $C_3^=$ = 15 mol s^{-1}, N_2 = 85 mol s^{-1}
Feed side pressure; 100 kPa
Permeate side pressure; 0.1 kPa
$C_3^=$ permeance = 1.0 x 10^{-7} mol m^{-2} s^{-1} Pa^{-1}
N_2 permeance = 5.6 x 10^{-10} mol m^{-2} s^{-1} Pa^{-1}
Permeance ratio = 180
Membrane temperature = 393 K
Number of cells = 5300

Fig. S3 shows the purity of propylene permeated as a function of the propylene recovery ratio. The purity of propylene permeated decreased with increasing recovery ratio. For example, the propylene purity at the propylene recovery ratios of 0.50, 0.70, and 0.90 were 0.96, 0.95, and 0.92, respectively.

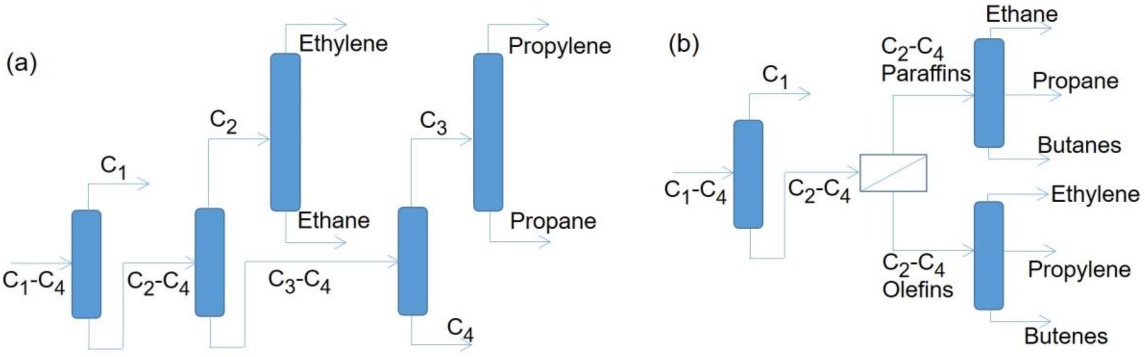

Figure S1. Olefin purification system from C_2-C_4 hydrocarbon mixture. (a) conventional process and (b) novel process including olefin concentration membrane.

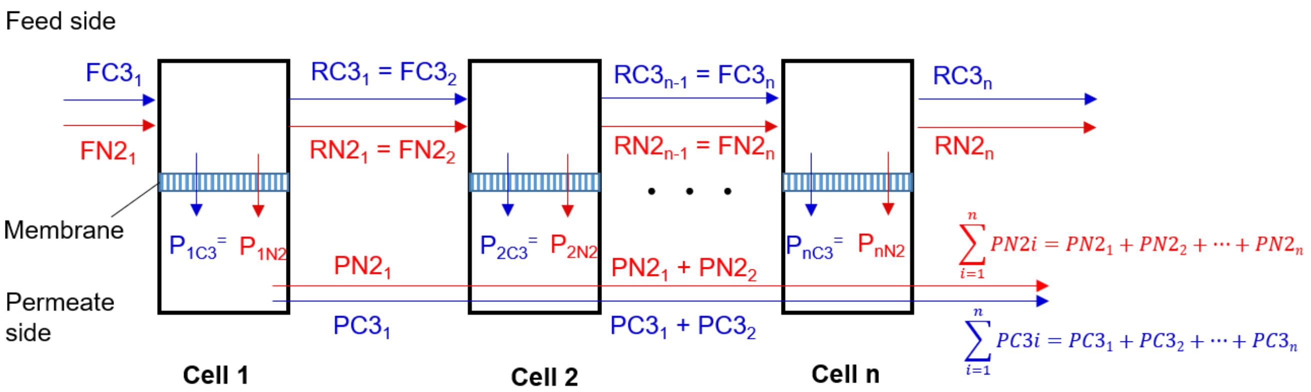

Figure S2. A model of calculation for $C_3^{=}/N_2$ mixture separation using Ag-*BEA membrane.

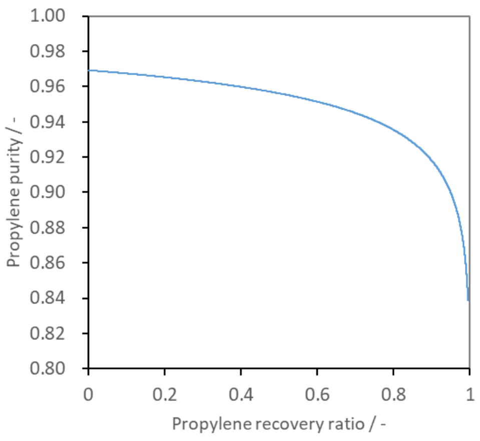

Figure S3. The purity of propylene permeated as a function of the propylene recovery ratio in propylene/nitrogen separation by Ag-*BEA membrane.

References

[1] M. Sakai, N. Fujimaki, G. Kobayashi, N. Yasuda, Y. Oshima, M. Seshimo, M. Matsukata, *Microporous Mesoporous Mater.* **2019**, *284*, 360-365.

[2] Y. Kamimura, W. Chaikittislip, K. Itabashi, A. Shimojima, T. Okubo, *Chem. Asian J.* **2010**, *5*, 2182-2191.

Author Contributions

M. Sakai designed the study, and wrote the initial draft of the manuscript. Y. Tuzuki and N. Fujimaki contributed to data collection and interpretation. All authors have given approval to the final version of the manuscript.
